# Impact of Pharmacogenetics on Pharmacokinetics of First-Line Antituberculosis Drugs in the HIRIF Trial

**DOI:** 10.1093/infdis/jiaf195

**Published:** 2025-04-17

**Authors:** Elizabeth Mackay, Gareth Platt, Charles A Peloquin, Meredith B Brooks, Julia M Coit, Gustavo E Velásquez, Henry Pertinez, Dante Vargas, Epifanio Sánchez, Roger I Calderón, Judith Jiménez, Karen Tintaya, Dalicxa Garcia, Elna Osso, Leo Lecca, Carole Mitnick, Geraint R Davies

**Affiliations:** Department of Clinical Infection, Microbiology and immunology, University of Liverpool, Liverpool, United Kingdom; Department of Clinical Infection, Microbiology and immunology, University of Liverpool, Liverpool, United Kingdom; Department of Pharmacotherapy and Translational Research, University of Florida, Gainesville, Florida, USA; Department of Global Health, Boston University School of Public Health, Boston, Massachusetts, USA; Deparment of Global Health and Social Medicine, Harvard Medical School, Boston, Massachusetts, USA; Department of Medicine, University of California, San Francisco, California, USA; Department of Clinical Infection, Microbiology and immunology, University of Liverpool, Liverpool, United Kingdom; Department of Pneumonology, Hospital Nacional Hipolito Unanue, Lima, Peru; Department of Pneumonology, Hospital Nacional Sergio Bernales, Lima, Peru; Advanced Research and Health, Lima, Peru; Socios en Salud, Lima, Peru; Socios en Salud, Lima, Peru; Socios en Salud, Lima, Peru; Deparment of Global Health and Social Medicine, Harvard Medical School, Boston, Massachusetts, USA; Deparment of Global Health and Social Medicine, Harvard Medical School, Boston, Massachusetts, USA; Socios en Salud, Lima, Peru; Deparment of Global Health and Social Medicine, Harvard Medical School, Boston, Massachusetts, USA; Department of Clinical Infection, Microbiology and immunology, University of Liverpool, Liverpool, United Kingdom

**Keywords:** tuberculosis, pharmacokinetics, pharmacogenetics, rifampicin, isoniazid, *NAT2*, *SLCO1B1*

## Abstract

**Background:**

Variability in the pharmacokinetics (PK) of first-line antituberculosis drugs (rifampicin [RIF], isoniazid [INH], and pyrazinamide [PZA]) is high and may be influenced by pharmacogenetic polymorphism. We performed a pharmacogenetic substudy in 90 participants with PK data from the HIRIF trial in Peru.

**Methods:**

Relevant single-nucleotide polymorphisms (SNPs) in the *NAT2, SLCO1B1*, *AADAC,* and *AOX1* loci were genotyped using real-time polymerase chain reaction (PCR).

**Results:**

The proportions of slow, intermediate, and fast acetylators predicted by a conventional 6-SNP *NAT2* panel were 32.5%, 48.2%, and 19.2%, respectively. A single *NAT2* tag SNP (rs1495741) agreed with the panel-predicted phenotype in 91% and was a better predictor of INH area under the curve (AUC). Accounting for discrepancies possibly caused by rare alleles not represented in the panel or that could be unequivocally resolved using observed AUC, sensitivity of the tag SNP was 97.7%. A previously described SNP in *SLCO1B1* (rs4149032) was present at an allele frequency of 0.31 and appeared to influence RIF AUC and maximum concentration (C_max_) at a dose of 20 mg/kg, despite an extreme distribution of alleles across the randomized arms. The *AADAC* SNP (rs1803155) predominated in the study population and was not linked to RIF PK, although an effect could have been missed due to sample size and allele frequency. There was no association between PZA PK and a common SNP in *AOX1* (rs55754655).

**Conclusions:**

A tag SNP approach may offer simpler and cheaper prediction of INH PK. Further exploration of the impact of *SLCO1B1* SNPs on RIF PK is required in this and other populations.

**Clinical Trials Registration**. NCT01408914.

## Teaser

Single-nucleotide polymorphisms (SNPs) in *SLCO1B1* and *AADAC* did not predict pharmacokinetics of rifampicin in a dose-ranging trial. A single SNP in *NAT2* may merit further evaluation as a means of identifying people at higher risk of side effects.

First-line treatment of tuberculosis requires prolonged administration of multidrug therapy for at least 6 months and has changed little in the last 50 years. The duration and complexity of treatment are important barriers to addressing the public health challenge of tuberculosis, which remains the single biggest killer amongst bacterial pathogens [1]. Novel agents developed to date have not supplanted the standard 3 core drugs, rifampicin (RIF), isoniazid (INH), and pyrazinamide (PZA), while recent research has also focused on outstanding questions in optimizing the clinical use of these older agents [2]. Although licensed for many decades, a deeper understanding of the pharmacology of first-line drugs may offer opportunities for improved efficacy and safety of tuberculosis treatment.

Pharmacokinetic-pharmacodynamic considerations suggest that higher doses of some components of the first-line regimen, such as RIF, could contribute to improved outcomes for all patients and pivotal trials are now evaluating this question despite initial concerns about safety, particularly drug-induced liver injury [3, 4]. Stratified or personalized approaches to treatment of drug-sensitive tuberculosis have also been proposed: The pharmacokinetics (PK) of INH are strongly determined by the highly polymorphic *NAT2* gene [5] and precision dosing approaches may be associated with fewer adverse events and improved outcomes in Asian populations [6, 7]. The impact of variation caused by single nucleotide polymorphisms (SNPs) in key loci controlling drug metabolism and distribution of other first-line drugs is currently less clear.

Like INH, RIF PK exhibits high interindividual variability and the drug causes dramatic autoinduction of its own metabolism, with plasma area under the curve (AUC) falling by approximately 30% in the first weeks of treatment [8]. However, while it is a powerful inducer, RIF is not itself a significant substrate of microsomal cytochrome P450 (CYP) enzyme systems and there have been conflicting reports on the impact of the hepatic organic anion uptake transporter SLCO1B1 [9–16] and the hepatic esterase AADAC on disposition of RIF and the alternate rifamycin rifapentine [17–19]. The pharmacogenetics of PZA metabolism also remain largely uncharacterized, although the enzyme xanthine oxidase, encoded by the polymorphic *AOX1* locus, appears to be an important pathway in its metabolic schema [20].

The HIRIF trial studied higher doses of RIF, up to 20 mg/kg, within the current first-line regimen in Peru, reporting faster bacillary elimination and similar rates of adverse events compared to the standard dose [21]. A comprehensive pharmacokinetic substudy of the trial found that interindividual variability in plasma PK of RIF and INH among participants was high [22]. While the pharmacogenetic determinants of this PK variability have been studied in an African and Asian context, limited information exists on their distribution in Latin America [9–19, 23–25]. We performed a substudy of the HIRIF trial to further investigate the contribution of pharmacogenetics to PK outcomes in Peru, particularly at higher dose of RIF.

## METHODS

HIRIF was a phase 2B randomized trial comparing RIF doses of 10, 15, and 20 mg/kg/day (delivered with standard weight-based doses of companion drugs) during the intensive phase of tuberculosis treatment (NCT01408914). Adults with newly diagnosed, previously untreated, smear positive (≥ 2+) pulmonary tuberculosis were recruited in Lima, Peru. All study procedures, including the separate PK and pharmacogenetics informed consent processes, were approved by the sponsors’ and local ethical oversight bodies [21].

Plasma concentrations of RIF, INH, and PZA were obtained using a hybrid intensive/sparse sampling scheme after 2 weeks of dosing. Drugs were assayed using validated Liquid Chromatography-Mass Spectrometry (LC-MS) methods at the University of Florida and summary PK parameters including the peak plasma concentration (C_max_) and area under the plasma concentration time curve (AUC_all_) were obtained by noncompartmental methods as previously described [22].

Whole blood was collected during the PK visit and frozen at −80°C. Genomic DNA was extracted at the University of Liverpool using the QIAamp Blood Midi kit (Qiagen). Genotyping was performed in duplicate by real-time polymerase chain reaction (PCR) for the following SNPs and loci: NAT2*5 (rs1801280), NAT2*6 (rs1799930), NAT2*7 (rs1799931), NAT2*12 (rs1208), NAT2*13 (rs1041983), NAT2*14 (rs1801279), a tag SNP located 14 kb 3′ of the *NAT2* exon (rs1495741), *SLCO1B1* (rs4149032), *AADAC* (rs1803155), and *AOX1* (rs55754655). Briefly, 23 μL of PCR master mix was placed in each of the central wells of a 96-well white PCR plate (MJ) comprising the following: 12.5 μL ABgene qPCR mix MM, 9.25 μL double-distilled water (Sigma-Aldrich), and 1.25 μL primer/probe mix. Then, 2 μL of sample genomic DNA at 20 ng/mL (or 2 μL of double distilled water for blanks) was added to each well and the plate sealed with a clear membrane before spinning for 10 seconds at 1000*g*. PCR amplification was performed in an Opticon 2.0 thermal cycler (Bio-Rad Laboratories) with an initial cycle of 95°C for 10 minutes (denaturation/enzyme activation step) followed by 49 cycles of 15 seconds at 92°C and 90 seconds at 60°C (combined annealing/extension step). Genotypes were analyzed using MJ Opticon Monitor analysis software using the cycle threshold values for the dyes FAM and VIC corresponding to the different alleles.

Predictions of acetylator phenotype were based on genetic diplotype according to the number of mutant loss of function alleles present (*5, *6, *7, or *14): fast (no mutant alleles), intermediate (1 mutant allele), and slow (2 mutant alleles). Linkage analysis and analysis of variance models were performed in R version 3.4.4/Bioconductor. PK parameters were analyzed on a logarithmic scale and nested linear models using a weight-adjusted dose metric and different genetic models were compared using the Akaike information criterion (AIC), likelihood ratio test and Wald tests of the coefficients.

## RESULTS

Genotyping was performed on 90 participants in the HIRIF trial who had consented to genetic analysis and had paired PK data available unaffected by study halts. All SNPs genotyped were in Hardy-Weinberg equilibrium.

**Figure 1.**
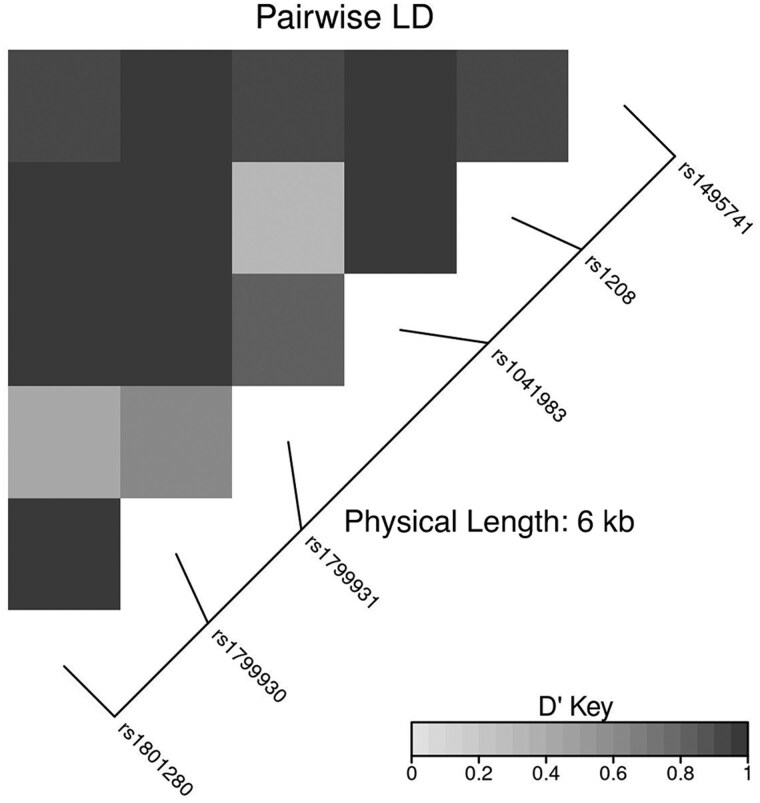
Linkage disequilibrium (LD) heatmap for *NAT2* alleles.

The mutant SNP allele frequencies for the 6 SNP *NAT2* panel were as follows: NAT2*5 (rs1801280) 0.23, NAT2*6 (rs1799930) 0.09, NAT2*7 (rs1799931) 0.27, NAT2*12 (rs1208) 0.22, NAT2*13 (rs1041983) 0.37, and NAT2*14 (rs1801279) 0.00. The allele frequency for the *NAT2* tag SNP (rs1495741) was 0.41. As expected, the 7 *NAT2* SNPs were in strong linkage disequilibrium, particularly the tag SNP which had a D′ value of 0.91 or greater for all other *NAT2* SNPs (Figure 1). Because no mutants were detected for the NAT2*14 (rs1801279) allele in the study population, it was not considered further in our analysis.

**Figure 2.**
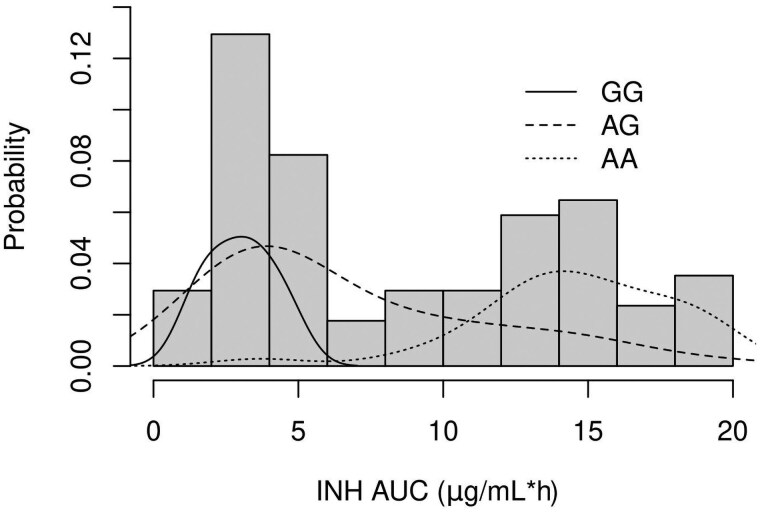
Distribution of isoniazid (INH) area under the curve (AUC) with superimposed nonparametric scaled density estimates for rs1495741 genotypes (*NAT2* tag single-nucleotide polymorphism).

The distribution of INH AUC exhibited at least 2 prominent modes as expected (Figure 1). Conventional genotypic predictions of the proportions of slow, intermediate, and fast INH acetylators using the 6-SNP panel were 32.5%, 48.2%, and 19.2%, respectively. Concordance between the 6-SNP panel and the tag SNP was generally high with disagreement in only 8 participants (8.9%) (Table 1). Notably, 3 of these discrepancies were predicted as fast acetylators by the 6-SNP panel but as slow acetylators by the tag SNP. In all of these participants, the observed INH AUC was greater than 12 μg/mL*h, clustering close to the mean AUC of the slow acetylator group (13.7 μg/mL*h) rather than that of the fast acetylator group (5.2 μg/mL*h) and lying beyond the predicted distribution of AUC for fast acetylators obtained by either genotyping method (Figure 2). These pharmacokinetic exposures appear clearly consistent with a slow acetylator phenotype, suggesting that the panel prediction was incorrect. The other 5 discordant predictions involved discrepancies only of a single allele and were distributed symmetrically in the confusion matrix, although 3 could also be resolved unequivocally in favor of the tag SNP based on their AUC, giving an overall sensitivity of 97.7%.

**Table 1.** Confusion Matrix for Tag SNP (rs1495741) Versus 6-SNP Panel Predicted Acetylator Phenotype

	Fast	Intermediate	Slow
AA	3	1	25
AG	0	37	2
GG	13	2	0

Abbreviation: SNP, single-nucleotide polymorphism.

In analysis of variance models, the tag SNP predicted the INH AUC significantly better than the conventional 6-SNP method in the study population (Table 2) and this advantage remained even when the 3 widely discrepant predictions were excluded from the model (AIC −2.176 and 9.004 for the tag SNP and panel, respectively). Both variables were much stronger predictors of INH AUC than the weight-adjusted INH dose itself. The conclusions of the analysis using C_max_ as a measure of PK exposure instead of AUC were similar.

**Table 2.** One-Way ANOVA of Performance of the Tag SNP (rs1495741) Versus 6-SNP Panel in Predicted INH AUC_0–6_ and C_max_

Model	Point Estimate	95% CI	Wald Test *P* Value	AIC
AUC_all_, μg/mL*h				
Base model	Intercept 6.711	5.665–7.950	<.001	60.436
SNP panel	Fast 3.721	2.725–5.081	<.001	26.841
	Intermediate 5.487	3.795–7.932	.042	
	Slow 12.829	8.659–19.007	<.001	
rs1495741	AA 13.962	11.544–16.887	<.001	−5.726
	AG 5.479	4.262–7.043	<.001	
	GG 2.760	1.992–3.821	<.001	
INH dose, mg/kg	per mg/kg −0.231	−.161 to −.331	.870	63.093
C_max_, μg/mL				
Base model	Intercept 2.157	1.902–2.447	<.001	11.204
SNP panel	Fast 1.430	1.127–1.813	.004	−17.841
	Intermediate 1.866	1.408–2.473	.067	
	Slow 3.412	2.527–4.607	<.001	
rs1495741	AA 3.628	3.122–4.215	<.001	−45.067
	AG 1.863	1.528–2.271	<.001	
	GG 1.156	.894–1.495	<.001	
INH dose, mg/kg	Per mg/kg 0.180	.051–.253	.222	11.666

Abbreviations: AIC, Akaike information criterion; ANOVA, analysis of variance; AUC, area under the curve; CI, confidence interval; C_max_, maximum concentration; INH, isoniazid; SNP, single-nucleotide polymorphism.

The mutant allele frequency for *SLCO1B1* (rs4149032) was 0.31. Although this SNP was relatively common, it was not distributed evenly across the trial arms. The frequencies of the mutant allele in the 10, 15, and 20 mg/kg dose groups were 0.31, 0.48, and 0.80, respectively, with 50% of those carrying mutant alleles in the highest dose group. The probability of this distribution of alleles having occurred during randomization by chance was 1 in 416 (Fisher exact test *P* = .002) but seems the only explanation, given the lack of any possible causal association between assignment to trial arm and *SLCO1B1* genotype. This correlation created substantial collinearity with trial arm and therefore with RIF dose in exploring the impact of the 2 variables.

**Figure 3.**
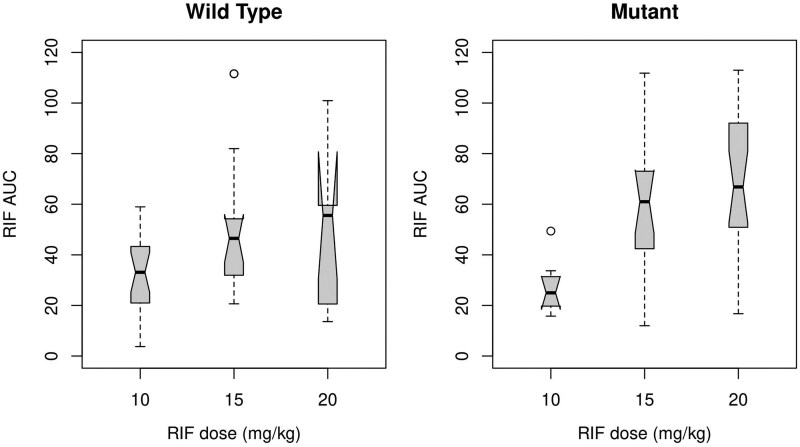
Rifampicin (RIF) plasma area under the curve (AUC) by dose level, stratified by *SLCO1B1* genotype (rs4149032), wild type (left) and mutant (right), using a dominant genetic model. Boxplots show median, interquartile range, range (whiskers) and any outliers.

Exploratory analysis supported empirical choice of a dominant genetic model for this locus and suggested that the relationship of RIF AUC and C_max_ to dose was much stronger in an analysis stratified by the rs4149032 SNP, with a possible threshold effect for doses above 10 mg/kg (Figure 3). Indeed, the impact of RIF weight-adjusted dose was inconsistent and of borderline statistical significance in wild-type participants but was consistent and strongly significant in participants with any *SLCO1B1* mutation. While in univariate analysis both trial arm and rs4149032 were associated with measures of RIF PK exposure, only the former was retained in the final model. However, when the dose was instead categorized at a threshold of 20 mg/kg there was some evidence of an impact of rs4149032 mutants, which was comparable in magnitude to a doubling of RIF dose. Although this analysis was thus supportive of an independent effect of the SNP at higher doses of RIF, all full interaction models fitted resulted in a substantial rise in AIC, often with unstable coefficients suggesting over-parameterization (Table 3).

**Table 3.** Two-Way ANOVA of RIF dose and the *SLCO1B1* SNP rs4149032

Model	Point Estimate	95% CI	Wald Test *P* Value	AIC
AUC_all_, μg/mL*h				
Base model	Intercept 41.805	36.247– 48.215	<.001	34.628
Trial arm	10 mg/kg 26.298	21.151–32.695	<.001	14.089
	15 mg/kg 47.970	35.436–64.936	<.001	
	20 mg/kg 58.028	45.536–79.182	<.001	
Dose 20 mg/kg	<20 mg/kg 35.887	30.468–42.270	<.001	26.488
	20 mg/kg 58.028	43.400–77.586	.002	
rs4149032, dominant model	Wild type 34.459	28.162–42.163	<.001	30.153
	Mutant 50.200	37.984–66.342	.010	
Trial arm + rs4149032	10 mg/kg 25.223	20.003–31.805	<.001	15.235
	15 mg/kg 45.186	33.063–61.756	<.001	
	20 mg/kg 51.796	36.885–72.736	<.001	
	Mutant (increase) 3.966	2.169–6.623	.301	
Dose 20 mg/kg + rs4149032	10 mg/kg/WT 32.531	26.598–39.788	<.001	26.226
	20 mg/kg/WT 47.751	35.053–65.049	.017	
	Mutant (increase) 9.266	4.707–16.019	.093	
C_max_, μg/mL)				
Base model	Intercept 8.946	7.858–10.185	<.001	18.42
Trial arm	10 mg/kg 6.003	4.909–7.340	<.001	0.614
	15 mg/kg 10.026	7.634–13.167	<.001	
	20 mg/kg 11.922	8.947–15.887	<.001	
Dose 20 mg/kg	<20 mg/kg 7.826	6.740–9.089	<.001	11.049
	20 mg/kg 11.922	9.145–15.543	.003	
rs4149032	Wild type 7.384	6.153–8.861	<.001	13.164
	Mutant 10.679	8.301–13.740	.005	
Trial arm + rs4149032	10 mg/kg 5.706	4.278–7.610	<.001	1.548
	15 mg/kg 9.221	6.743–12.609	.002	
	20 mg/kg 10.386	8.060–13.383	<.001	
	Mutant (increase) 1.107	1.009–1.169	.174	
Dose 20 mg/kg + rs4149032	10 mg/kg 7.035	5.859–8.448	<.001	10.065
	20 mg/kg 9.713	7.336–12.862	.027	
	Mutant (increase) 2.121	1.182–3.457	.053	

Abbreviations: AIC, Akaike information criterion; ANOVA, analysis of variance; AUC, area under the curve; CI, confidence interval; RIF, rifampicin; SNP, single-nucleotide polymorphism.

The *AADAC* rs1803155 SNP was present at an allele frequency of 0.86 . It was not associated with changes in RIF C_max_ and AUC and there was no significant statistical interaction with RIF dose or with the *SLCO1B1* rs4149032 SNP. The allele frequency for the *AOX1* SNP (rs55754655) in the study population was low at 0.03 and was not associated with changes in the pharmacokinetic parameters of PZA.

## DISCUSSION

While the impact of the highly polymorphic *NAT2* locus on the pharmacokinetics of INH has long been recognized, there are relatively few data on the distribution of *NAT2* alleles in patients with tuberculosis in Latin America [23–25]. By contrast, there have been fewer and apparently conflicting reports on the pharmacogenetic basis of the high interindividual PK variability of RIF, with both the *SLCO1B1* and *AADAC* loci possibly implicated in studies from different geographical areas [9–19]. We sought to address these questions in this substudy of Peruvian patients with tuberculosis randomized to 3 dose levels of RIF during first-line treatment in a clinical trial and who also underwent PK sampling [21, 22].

Mutant *NAT2* alleles associated with slower acetylation were common in our cohort resulting in a predicted proportion of fast acetylators of 19.2%, similar to recent reports in adult patients with tuberculosis from Peru [25], Bolivia and Argentina [24], and in children with tuberculosis in Venezuela [23]. This proportion is lower than studies in various Native American populations [24] and likely reflects the overall impact of historical genetic admixture from Europe and Asia in the Peruvian population.

The diverse *NAT2* alleles are known to form a haploblock with the tag SNP rs1495741, located 14 kb 3′ of the *NAT2* exon, suggesting that this single SNP may be capable of adequately representing the genetic variation captured by the traditional 6- or 7-SNP panel [26]. We found the tag SNP to be highly concordant with the predictions of the 6-SNP panel and to be more accurate in cases of extreme discrepancy, which could reflect the presence in the population of rare mutant alleles not captured by the panel. These findings are in agreement with those of previous studies, including that of Chamorro et al who claimed a sensitivity of 97% and specificity of 99.3% of the tag SNP for prediction of slow acetylator phenotype in a Latin American context [27]. This is the group of patients at highest risk of drug-induced liver injury whilst on tuberculosis treatment [28] who may benefit most from stratified approaches to dosing of INH. While it may be possible to improve performance using more sophisticated bioinformatic approaches to SNP panels [29] or targeted sequencing, this simpler approach may be sufficiently accurate for clinical purposes and worthy of further evaluation.

The metabolism of RIF is complex and dynamic due to saturable autoinduction of metabolism and nonlinear pharmacokinetics at doses higher than 10 mg/kg [3, 6]. RIF is a substrate for the hepatic organic anion uptake transporter OATP1B1 [30] but may also directly inhibit uptake of other substrates [31], possibly through competitive mechanisms. OATP1B1 is coded by the gene locus *SLCO1B1* in which at least 14 nonsynonymous SNPs have been identified, 6 of which have been characterized as conferring loss of function [32]. Such mutations would be expected to reduce accessibility of RIF to hepatic clearance and to be associated with higher plasma PK exposures.

rs4149032 is an SNP in *SLCO1B1* previously linked to lower RIF exposure in some African populations [9–11]. However, this association has not been confirmed in other studies from Africa [13–16] and India [12]. We did not observe such an association in our cohort in Peru. This may be because rs4149032 is an intronic SNP and in variable linkage disequilibrium with nonsynonymous SNPs such as rs11045819 (*4) and rs4149056 (*5) in different populations. The haplotypic structure of *SLCO1B1* is complex with relatively large haploblocks, which may be variably captured by proposed tag SNPs in different populations [33]. Hence the performance of rs4149032 as a tag SNP may be population dependent, varying with the local allele frequencies of the different SNPs and their correlation. Few studies to date have simultaneously characterized all the relevant SNPs in *SLCO1B1*, focusing on previously identified candidate SNPs in isolation. However, other factors might also have an impact, including the relatively small sample size in most of the studies, precision of the PK parameter estimates obtained, and timing of samples in relation to autoinduction of metabolism.

Our study is the first to our knowledge to examine the impact of *SLCO1B1* SNPs in the context of higher doses of RIF than are conventionally used. Plasma AUC of RIF appears to be nonlinear with doses above 10 mg/kg [3] and saturability of hepatic uptake mechanisms is one possible explanation of this observation, a hypothesis on which the HIRIF trial could potentially provide information. Although we observed a greater increase in RIF AUC in the presence of any *SLCO1B1* mutation at higher doses of RIF, interpretation of this finding was complicated by the chance occurrence of an extreme distribution of mutant alleles across the treatment arms. Hence, we can only speculate that, while participants with wild-type OATP1B1 may have retained additional capacity for RIF transport at higher doses, those with *SLCO1B1* loss-of-function mutations might be expected to have no such reserve and thus greater increases in plasma exposure of RIF. Hence, the nonlinearity and interindividual variability of RIF PK at higher doses could be influenced importantly by polymorphism in *SLCO1B1,* and should be studied in more detail in a wider range of populations.

Although it has long been suggested that the primary metabolic pathway for rifamycins in humans involves hepatic esterases, more recent detailed in vitro studies have proposed that they are substrates for arylacetamide deacetylase (AADAC), a microsomal serine deacetylase, rather than the better characterized carboxylesterases (CES-1/2), which are responsible for activation of numerous ester prodrugs [34]. Two studies have associated lower clearance and higher plasma exposure of the related rifamycin rifapentine with the rs1803155 SNP in *AADAC* at 2 different dose levels [17, 18] and another study reported similar findings for RIF [19] at the standard dose [12]. We did not confirm any change in RIF exposure with this SNP across any of the doses studied and this analysis did not suffer from the difficulties caused by the extreme distribution of rs4149032 alleles. The primary metabolites of RIF and rifapentine are formed by esterase attack at the same functional group (25 position) so this finding is perhaps unexpected. It is notable that these prior studies almost exclusively included people of African heritage and that in the few non-African participants AADAC was not associated with rifapentine exposure [18]. However, in agreement with another recent study from Peru [25], the rs1803155 mutant allele frequency was higher in our study population (0.86) than in these previous studies, which ranged from 0.70 to 0.82, and the sample size was smaller. These factors would make it difficult to detect any effect, particularly under a dominant genetic model, because of the rarity of wild-type homozygotes. Larger studies in other populations, where the mutant allele frequency is lower, may be required to clarify the impact of *AADAC* polymorphism on PK of rifamycins.

Although the xanthine oxidase pathway has been implicated in the metabolic schema of PZA the *AOX1* locus has not previously been explored as a possible source of PK variability of the drug. We examined only one common candidate SNP, which proved to be uncommon in the trial population so this question may be worthy of further study in other populations.

Although our study was conducted under clinical trial conditions and high-quality pharmacokinetic and genotyping data were available at a range of RIF doses, our conclusions were limited by the relatively small sample size of the substudy and our inability to extensively characterize the haplotypic structure of the *SLCO1B1* and *AADAC* loci.

We have shown that the proportion of fast acetylators is relatively low in patients with tuberculosis in Lima and that a single common tag SNP may offer a reliable and simple test to determine acetylator status in this population. We also suggest that SNPs in *SLCO1B1* may be implicated in the nonlinearity of RIF PK at higher doses and should be further studied in a wider range of settings.

## Notes

***Acknowledgment.*** Rifampicin for the HIRIF trial was donated by Sanofi-Aventis.

***Author contributions.*** E. M. and G. P. performed the genotyping and C. P. the pharmacokinetic analysis. G. D. and H. P. carried out the statistical analysis. C. M., L. L., G. V., D. V. V., E. G. S., M. B., J. C., E. O., D. G., K. T., J. J., and R. D. were members of the study team who executed the trial and prepared the data. All authors read and approved the final manuscript.

***Financial support.*** This work was supported by the US National Institutes of Health (grant numbers U01AI091429 to C. M. for the HIRIF trial, and K08AI141740 to G. V. for analysis).
